# A novel defined pyroptosis-related gene signature for predicting the prognosis of ovarian cancer

**DOI:** 10.1038/s41420-021-00451-x

**Published:** 2021-04-07

**Authors:** Ying Ye, Qinjin Dai, Hongbo Qi

**Affiliations:** 1 The Department of Obstetrics, The First Affiliated Hospital of Chongqing Medical University, Chongqing, 400016 China; 2 State Key Laboratory of Maternal and Fetal Medicine of Chongqing Municipality, Chongqing Medical University, Chongqing, 400016 China; 3 Guangzhou Women and Children’s Medical Center, Guangzhou Medical University, Guangzhou 510623, Guangdong, China

**Keywords:** Ovarian cancer, Transcriptomics

## Abstract

Ovarian cancer (OC) is a highly malignant gynaecological tumour that has a very poor prognosis. Pyroptosis has been demonstrated in recent years to be an inflammatory form of programmed cell death. However, the expression of pyroptosis-related genes in OC and their correlations with prognosis remain unclear. In this study, we identified 31 pyroptosis regulators that were differentially expressed between OC and normal ovarian tissues. Based on these differentially expressed genes (DEGs), all OC cases could be divided into two subtypes. The prognostic value of each pyroptosis-related gene for survival was evaluated to construct a multigene signature using The Cancer Genome Atlas (TCGA) cohort. By applying the least absolute shrinkage and selection operator (LASSO) Cox regression method, a 7-gene signature was built and classified all OC patients in the TCGA cohort into a low- or high-risk group. OC patients in the low-risk group showed significantly higher survival possibilities than those in the high-risk group (*P* < 0.001). Utilizing the median risk score from the TCGA cohort, OC patients from a Gene Expression Omnibus (GEO) cohort were divided into two risk subgroups, and the low-risk group had increased overall survival (OS) time (*P* = 0.014). Combined with the clinical characteristics, the risk score was found to be an independent factor for predicting the OS of OC patients. Gene ontology (GO) and Kyoto Encylopedia of Genes and Genomes (KEGG) analyses indicated that immune-related genes were enriched and that the immune status was decreased in the high-risk group. In conclusion, pyroptosis-related genes play important roles in tumour immunity and can be used to predict the prognosis of OCs.

## Introduction

Ovarian cancer (OC) is a common malignancy of the female reproductive system, second only to cervical cancer and uterine corpus cancer in terms of incidence. OC has extremely high recurrence and mortality rates, which seriously threaten women’s health. In the United States, ~22,530 new OC cases were diagnosed, and OC caused 13,980 deaths in 2019^1^. Due to the lack of effective screening tools and difficulties in early diagnosis, 80% of OC patients are already at an advanced stage when diagnosed, and 50–70% of patients will experience recurrence within 2 years after treatment, with a poor 5-year survival rate of 30%^2,3^. The current main treatments for OC are surgery and platinum-based chemotherapies. Despite recent improvements in treatments, the 5-year survival rate has been slow to improve^4^. Considering the limitations of OC treatments, new therapeutic targets are needed to improve the clinical outcome of OC; thus, reliable novel prognostic models are urgently required to make targeted therapies more feasible.

Pyroptosis, also known as cellular inflammatory necrosis, is a novel form of programmed cell death^5^. Pyroptotic cells are characterized by cellular swelling and many bubble-like protrusions. Under an electron microscope, pyroptotic cells can be seen to first form a large number of vesicles. After these vesicles form, pores form on the cell membrane, which ruptures and the contents flow out^6^. The gasdermin family is the main executor of pyroptosis and includes *gasdermin-A to gasdermin-E* and *pejvakin* (*PJVK* or *DFNB59*)^7^. Gasdermin family proteins can be sheared and multimerized, which leads to cleavage of the N-terminal and C-terminal junctional structural domains and release of activated N-terminal regions; these regions bind to membrane lipids, phosphatidylinositol, and cardiolipin and localize into the pores in the cell membrane^8,9^. Cellular gasdermin family proteins form 10 to 20 nm pores in the cell membrane, and cell contents are slowly released through the membrane pores and trigger amplified inflammatory responses. Cells gradually flatten, producing 1–5 μm apoptotic vesicle-like protrusions (scorched vesicles), and cells gradually swell until the plasma membrane ruptures, with features such as nuclear condensation and chromatin DNA breakage^10,11^. Pyroptosis was initially found to be a key mechanism for combating infection, and a growing number of studies suggest that it also plays an important role in the development of tumours. It has been reported that inflammatory vesicles, gasdermin proteins, and proinflammatory cytokines, which are key components of pyroptosis, are associated with tumourigenesis, invasion, and metastasis^12^. Dupaul-Chicoine et al.^13^ knocked out inflammatory vesicle-related genes (*NLRP3* and *CASP1*) in transgenic mice and found that they were more likely to develop colon cancers than mice with wild-type versions of the genes. In addition, unlike apoptosis, when pyroptosis occurs, a variety of danger-associated signalling molecules and cytokines are activated and released, accompanied by a strong inflammatory response and activation of the immune system^14^. A few studies have suggested that the potent proinflammatory effect of pyroptosis is connected to the regulation of the tumour immune microenvironment. Defective *GSDMD* expression was found to be accompanied by a significant decrease in the number and activity of CD8^+^ T lymphocytes^15^. A recent study also confirmed the critical role of pyroptosis in the antitumour function of NK cells^16^.

Given the existing findings, we know that pyroptosis plays an important role in the development of tumours and antitumour processes; however, its specific functions in OC have been less studied. Thus, we performed a systematic study to determine the expression levels of pyroptosis-related genes between normal ovarian and OC tissues, explore the prognostic value of these genes, and study the correlations between pyroptosis and the tumour immune microenvironment.

## Results

### Identification of DEGs between normal and tumour tissues

**Fig. 1:**
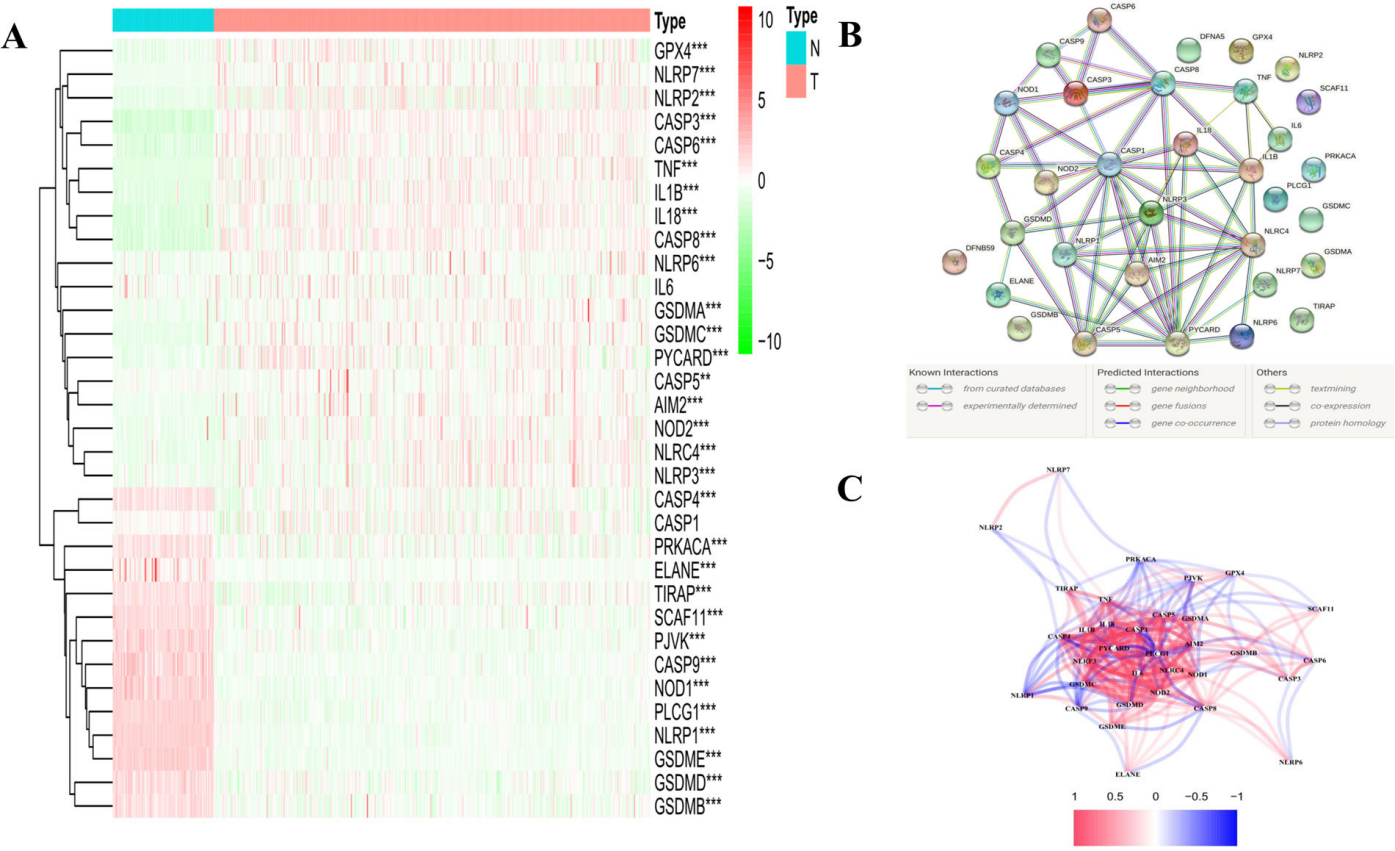
Expressions of the 33 pyroptosis-related genes and the interactions among them. **A** Heatmap (green: low expression level; red: high expression level) of the pyroptosis-related genes between the normal (N, brilliant blue) and the tumour tissues (T, red). *P* values were showed as: **P < 0.01; ***P < 0.001. **B** PPI network showing the interactions of the pyroptosis-related genes (interaction score = 0.9). **C** The correlation network of the pyroptosis-related genes (red line: positive correlation; blue line: negative correlation. The depth of the colours reflects the strength of the relevance).

The 33 pyroptosis-related gene expression levels were compared in the pooled Genotype-Tissue Expression (GTEx) and The Cancer Genome Atlas (TCGA) data from 88 normal and 379 tumour tissues, and we identified 31 differentially expressed genes (DEGs) (all *P* < 0.01). Among them, 13 genes (*PRKACA*, *GSDMB*, *SCAF11*, *PJVK*, *CASP9*, *NOD1*, *PLCG1*, *NLRP1*, *GSDME*, *ELANE*, *TIRAP*, *CASP4*, and *GSDMD*) were downregulated while 18 other genes (*GPX4*, *NLRP7*, *NLRP2*, *CASP3*, *CASP6*, *TNF*, *IL1B*, *IL18*, *CASP8*, *NLRP6*, *GSDMA*, *GSDMC*, *PYCARD*, *CASP5*, *AIM2*, *NOD2*, *NLRC4*, and *NLRP3*) were enriched in the tumour group. The RNA levels of these genes are presented as heatmaps in Fig. 1A (green: low expression level; red: high expression level). To further explore the interactions of these pyroptosis-related genes, we conducted a protein–protein interaction (PPI) analysis, and the results are shown in Fig. 1B. The minimum required interaction score for the PPI analysis was set at 0.9 (the highest confidence), and we determined that *CASP1*, *PYCARD*, *NLRC4*, *NLRP1*, *CASP5*, *NLRP3*, *CASP8*, and *AIM2* were hub genes. Among them, except for *CASP1*, other genes were all the DEGs between normal and tumour tissues. The correlation network containing all pyroptosis-related genes is presented in Fig. 1C (red: positive correlations; blue: negative correlations).

### Tumour classification based on the DEGs

**Fig. 2:**
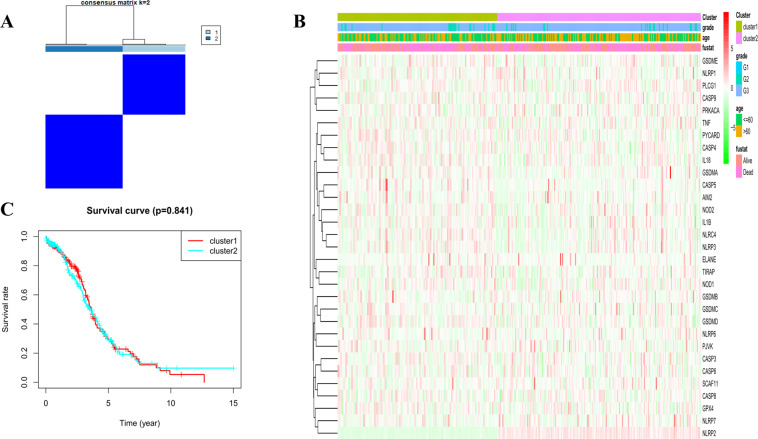
Tumour classification based on the pyroptosis-related DEGs. **A** 379 OC patients were grouped into two clusters according to the consensus clustering matrix (*k* = 2). **B** Heatmap and the clinicopathologic characters of the two clusters classified by these DEGs (G1, G2, and G3 are the degree of tumour differentiation. G1: High differentiated; G2: Moderate differentiated; G3: Poor differentiated). **C** Kaplan–Meier OS curves for the two clusters.

To explore the connections between the expression of the 31 pyroptosis-related DEGs and OC subtypes, we performed a consensus clustering analysis with all 379 OC patients in the TCGA cohort. By increasing the clustering variable (*k*) from 2 to 10, we found that when *k* = 2, the intragroup correlations were the highest and the intergroup correlations were low, indicating that the 379 OC patients could be well divided into two clusters based on the 31 DEGs (Fig. 2A). The gene expression profile and the clinical features including the degree of tumour differentiation (G1-G3), age (≤60 or >60 years) and survival status (alive or dead) are presented in a heatmap, but we found there’s little differences in clinical features between the two clusters (Fig. 2B). The overall survival (OS) time was also compared between the two clusters, but no obvious differences were found (*P* = 0.841, Fig. 2C).

### Development of a prognostic gene model in the TCGA cohort

**Fig. 3:**
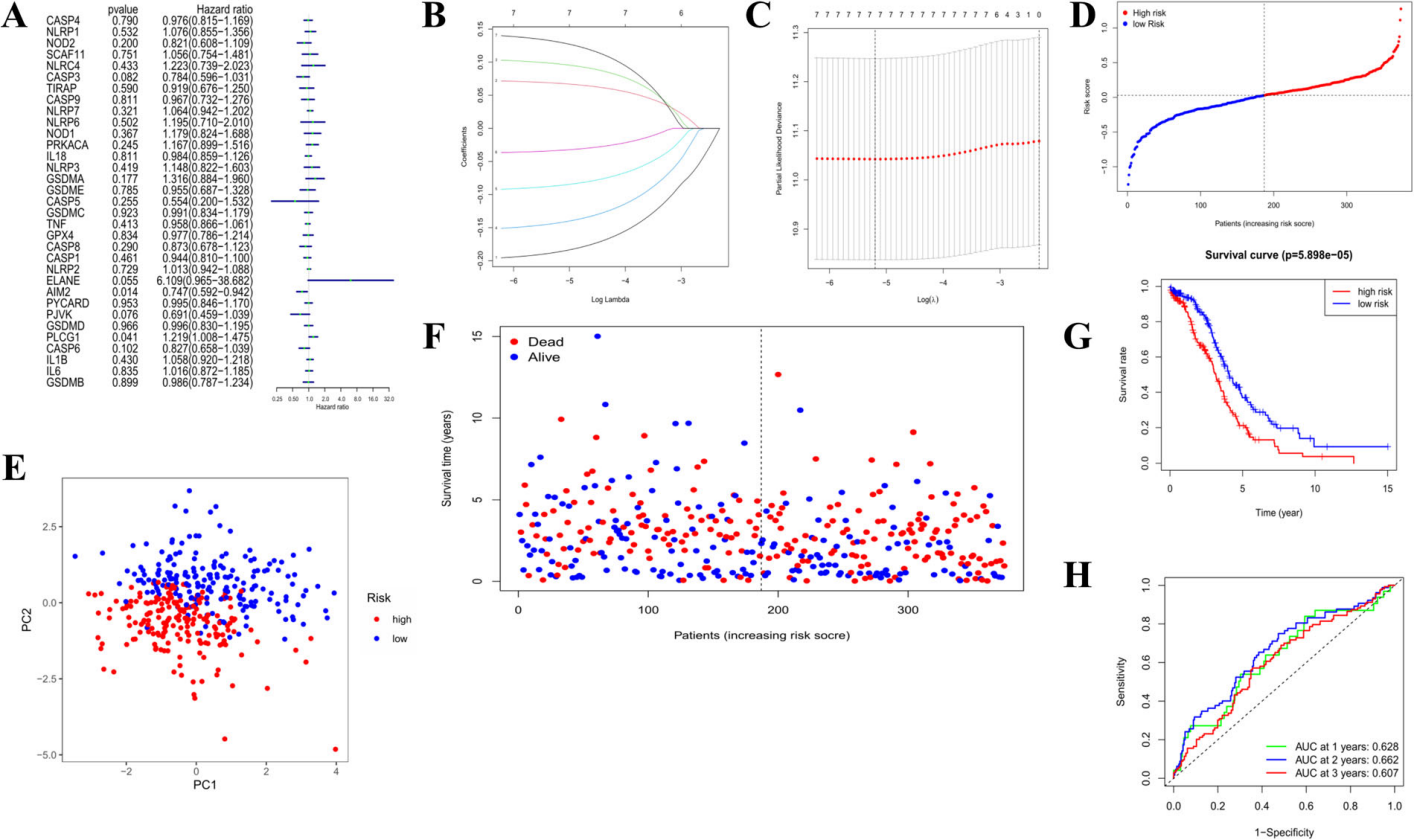
Construction of risk signature in the TCGA cohort. **A** Univariate cox regression analysis of OS for each pyroptosis-related gene, and 7 genes with *P* < 0.2. **B** LASSO regression of the 7 OS-related genes. **C** Cross-validation for tuning the parameter selection in the LASSO regression. **D** Distribution of patients based on the risk score. **E** PCA plot for OCs based on the risk score. **F** The survival status for each patient (low-risk population: on the left side of the dotted line; high-risk population: on the right side of the dotted line). **G** Kaplan–Meier curves for the OS of patients in the high- and low-risk groups. **H** ROC curves demonstrated the predictive efficiency of the risk score.

A total of 374 OC samples were matched with the corresponding patients who had complete survival information. Univariate Cox regression analysis was used for primary screening of the survival-related genes. The 7 genes (*AIM2*, *PLCG1*, *ELANE*, *PJVK*, *CASP3*, *CASP6*, and *GSDMA*) that met the criteria of *P* < 0.2 were retained for further analysis, and among them, 3 genes (*PLCG1*, *ELANE*, and *GSDMA*) were associated with increased risk with HRs >1, while the other 4 genes (*AIM2*, *PJVK*, *CASP3*, and *CASP6*) were protective genes with HRs <1 (Fig. 3A). By performing the least absolute shrinkage and selection operator (LASSO) Cox regression analysis, a 7-gene signature was constructed according to the optimum *λ* value (Fig. 3B, C). The risk score was calculated as follows: risk score = (−0.187*AIM2 exp.) + (0.068*PLCG1 exp.) + (0.097*ELANE exp.) + (−0.143*PJVK exp.) + (−0.086*CASP3 exp.) + (−0.033*CASP6 exp.) + (0.130*GSDMA exp.). Based on the median score calculated by the risk score formula, 374 patients were equally divided into low- and high-risk subgroups (Fig. 3D). The principal component analysis (PCA) showed that patients with different risks were well separated into two clusters (Fig. 3E). Patients in the high-risk group had more deaths and a shorter survival time than those in the low-risk group (Fig. 3F, on the right side of the dotted line). A notable difference in OS time was detected between the low- and high-risk groups (*P* < 0.001, Fig. 3G). Time-dependent receiver operating characteristic (ROC) analysis was applied to evaluate the sensitivity and specificity of the prognostic model, and we found that the area under the ROC curve (AUC) was 0.628 for 1-year, 0.662 for 2-year, and 0.607 for 3-year survival (Fig. 3H).

### External validation of the risk signature

**Fig. 4:**
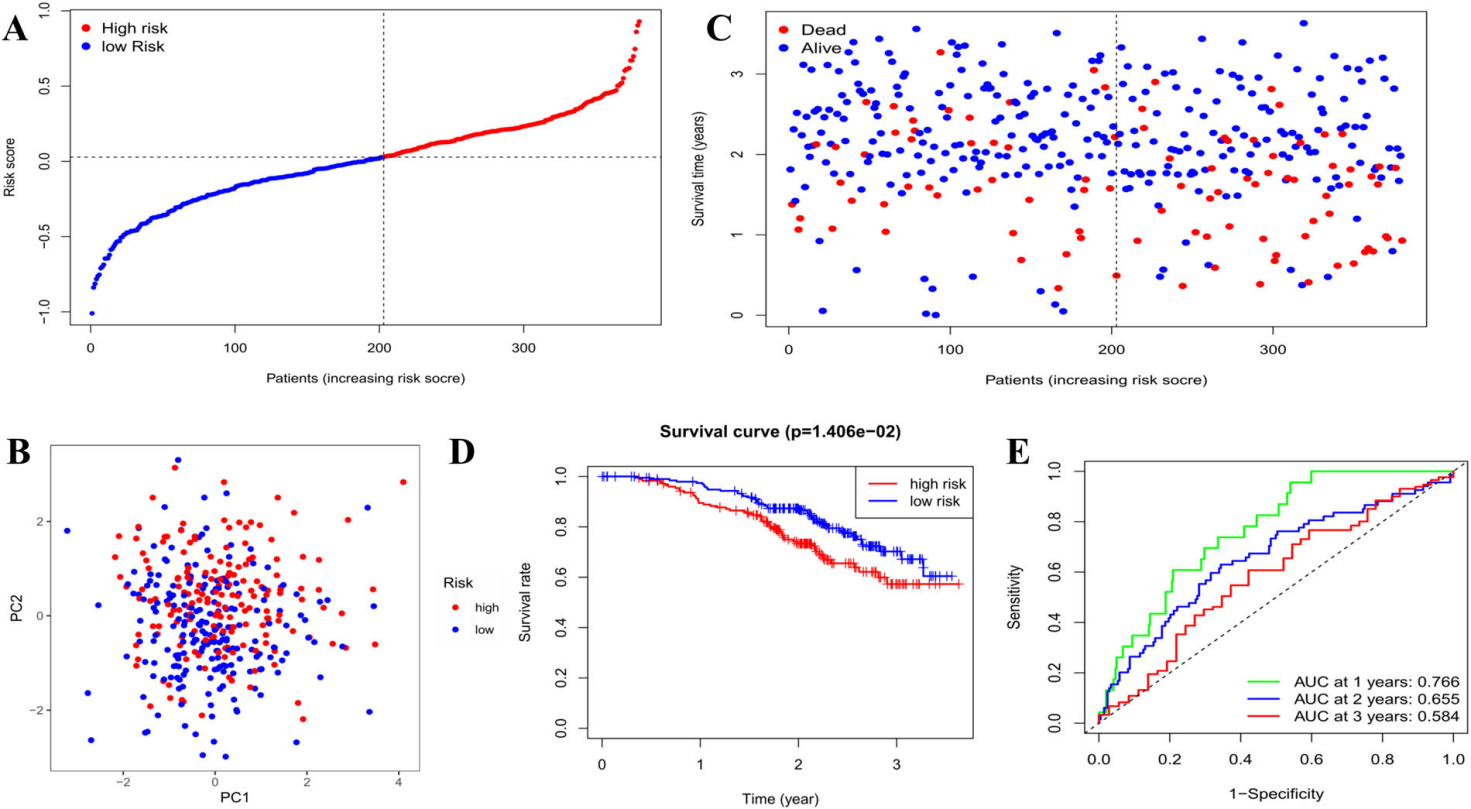
Validation of the risk model in the GEO cohort. **A** Distribution of patients in the GEO cohort based on the median risk score in the TCGA cohort. **B** PCA plot for OCs. **C** The survival status for each patient (low-risk population: on the left side of the dotted line; high-risk population: on the right side of the dotted line). **D** Kaplan–Meier curves for comparison of the OS between low- and high-risk groups. **E** Time-dependent ROC curves for OCs.

A total of 380 OC patients from a Gene Expression Omnibus (GEO) cohort (GSE140082) were utilized as the validation set. Before further analysis, the gene expression data were normalized by the “Scale” function. Based on the median risk score in the TCGA cohort, 203 patients in the GEO cohort were classified into the low-risk group, while the other 177 patients were classified into the high-risk group (Fig. 4A). The PCA showed satisfactory separation between the two subgroups (Fig. 4B). Patients in the low-risk subgroup (Fig. 4C, on the left side of the dotted line) were found to have longer survival times and lower death rates than those in the high-risk subgroup. In addition, Kaplan–Meier analysis also indicated a significant difference in the survival rate between the low- and high-risk groups (*P* = 0.014, Fig. 4D). ROC curve analysis of the GEO cohort showed that our model had good predictive efficacy (AUC = 0.766 for 1-year, 0.655 for 2-year, and 0.584 for 3-year survival) (Fig. 4E).

### Independent prognostic value of the risk model

**Fig. 5:**
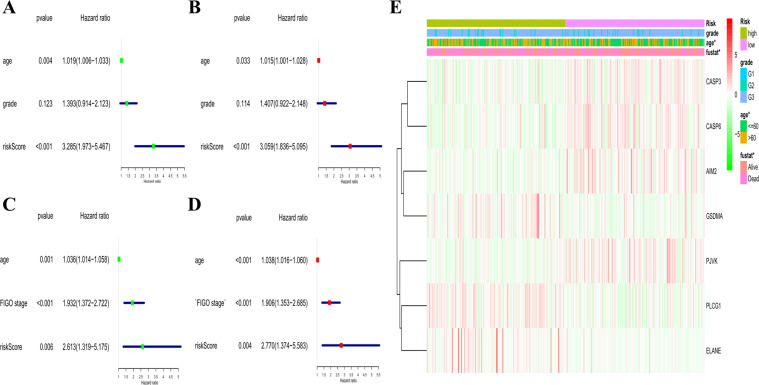
Univariate and multivariate Cox regression analyses for the risk score. **A** Univariate analysis for the TCGA cohort (grade: the degree of tumour differentiation, G1 to G3). **B** Multivariate analysis for the TCGA cohort. **C** Univariate analysis for the GEO cohort (FIGO stage: I to IV). **D** Multivariate analysis for the GEO cohort. **E** Heatmap (green: low expression; red: high expression) for the connections between clinicopathologic features and the risk groups (*P < 0.05).

We used univariate and multivariable Cox regression analyses to evaluate whether the risk score derived from the gene signature model could serve as an independent prognostic factor. The univariate Cox regression analysis indicated that the risk score was an independent factor predicting poor survival in both the TCGA and GEO cohorts (HR = 3.285, 95% CI: 1.973–5.467 and HR: 2.613, 95% CI: 1.319–5.175, Fig. 5A, C). The multivariate analysis also implied that, after adjusting for other confounding factors, the risk score was a prognostic factor (HR = 3.059, 95% CI: 1.836–5.095 and HR: 2.770, 95% CI: 1.374–5.583, Fig. 5B, D) for patients with OC in both cohorts. In addition, we generated a heatmap of clinical features for the TCGA cohort (Fig. 5E) and found that the age of patients and the survival status were diversely distributed between the low- and high-risk subgroups (*P* < 0.05).

### Functional analyses based on the risk model

**Fig. 6:**
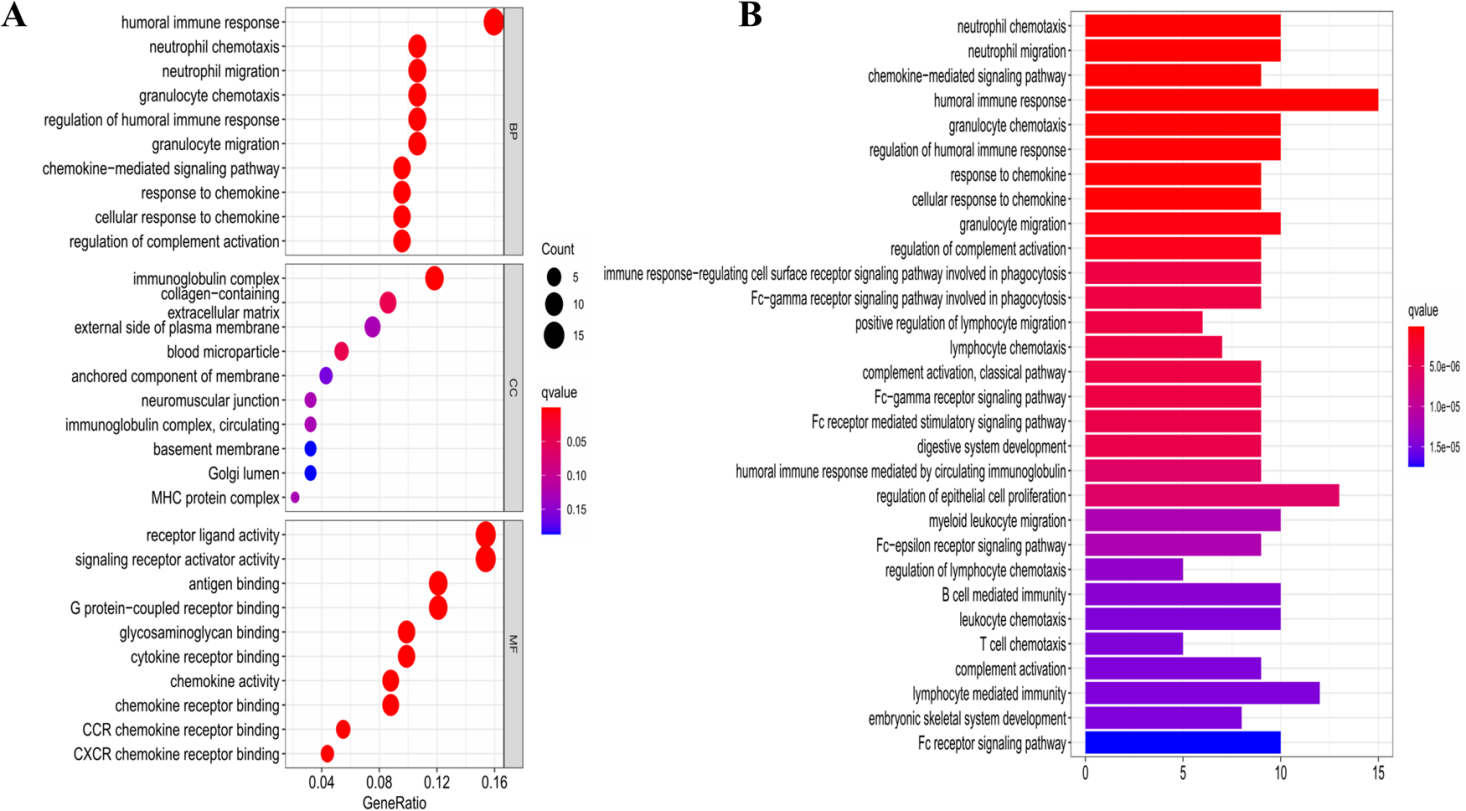
Functional analysis based on the DEGs between the two-risk groups in the TCGA cohort. **A** Bubble graph for GO enrichment (the bigger bubble means the more genes enriched, and the increasing depth of red means the differences were more obvious; q-value: the adjusted *p*-value). **B** Barplot graph for KEGG pathways (the longer bar means the more genes enriched, and the increasing depth of red means the differences were more obvious).

To further explore the differences in the gene functions and pathways between the subgroups categorized by the risk model, we utilized the “limma” R package to extract the DEGs by applying the criteria FDR < 0.05 and |log_2_FC | ≥ 1. In total, 115 DEGs between the low- and high-risk groups in the TCGA cohort were identified. Among them, 66 genes were upregulated in the high-risk group, while the other 49 genes were downregulated (the data are shown in Table S3). Gene ontology (GO) enrichment analysis and Kyoto Encyclopaedia of Genes and Genomes (KEGG) pathway analysis were then performed based on these DEGs. The results indicated that the DEGs were mainly correlated with the immune response, chemokine-mediated signalling pathways, and inflammatory cell chemotaxis (Fig. 6A, B).

### Comparison of the immune activity between subgroups

**Fig. 7:**
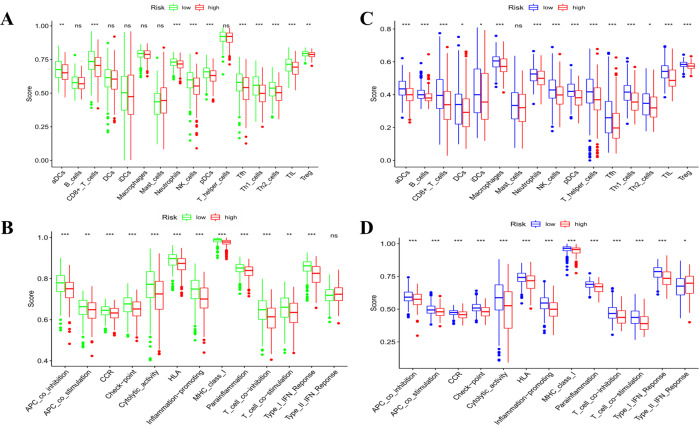
Comparison of the ssGSEA scores for immune cells and immune pathways. **A**, **B** Comparison of the enrichment scores of 16 types of immune cells and 13 immune-related pathways between low- (green box) and high-risk (red box) group in the TCGA cohort. **C**, **D** Comparison of the tumour immunity between low- (blue box) and high-risk (red box) group in the GEO cohort. *P* values were showed as: ns not significant; *P < 0.05; **P < 0.01; ***P < 0.001.

Based on the functional analyses, we further compared the enrichment scores of 16 types of immune cells and the activity of 13 immune-related pathways between the low- and high-risk groups in both the TCGA and GEO cohorts by employing the single-sample gene set enrichment analysis (ssGSEA). In the TCGA cohort (Fig. 7A), the high-risk subgroup generally had lower levels of infiltration of immune cells, especially of CD8^+^ T cells, neutrophils, natural killer (NK) cells, T helper (Th) cells (Tfh, Th1, and Th2 cells), tumour-infiltrating lymphocytes (TILs) and regulatory T (Treg) cells, than the low-risk subgroup. Except for the type-2 IFN response pathway, the other 12 immune pathways showed lower activity in the high-risk group than in the low-risk group in the TCGA cohort (Fig. 7B). When assessing the immune status in the GEO cohort, similar conclusions were drawn. In addition, we discovered that dendritic cells (DCs), induced dendritic cells (iDCs), and macrophages were enriched while type-2 IFN responses were downregulated in the low-risk group compared with the high-risk group (Fig. 7C, D).

## Discussion

In this study, we first studied the mRNA levels of 33 currently known pyroptosis-related genes in OC and normal tissues and found that most of them were differentially expressed. However, the two clusters produced by the consensus clustering analysis based on the DEGs did not show any significant differences in clinical characteristics. To further assess the prognostic value of these pyroptosis-related regulators, we constructed a 7-gene risk signature via Cox univariate analysis and LASSO Cox regression analysis, which was then validated to perform well in an external dataset. The functional analyses indicated that the DEGs between the low- and high-risk groups were related to immune-related pathways. The immune cell infiltration and activated pathways in the low- and high-risk groups were compared, and we found that the high-risk group had universally decreased levels of infiltrating immune cells and decreased activity of immune-related pathways compared with the low-risk group.

Pyroptosis, a novel form of programmed cell death, was found to play a dual-role in tumour development and therapeutic mechanisms in recent years. On the one hand, normal cells are stimulated by a large number of inflammatory factors released by pyroptosis, leading to their transformation into tumour cells^17^. On the other hand, the promotion of tumour cell pyroptosis could be a new therapeutic target^18^. In OC, how pyroptosis-related genes interact and whether they are related to the survival time of patients remain unknown. Our study generated a signature featuring 7 pyroptosis-related genes (*AIM2*, *PLCG1*, *ELANE*, *PJVK*, *CASP3*, *CASP6*, and *GSDMA*) and found that it could predict OS in OC patients. Absent in melanoma 2 (*AIM2*) was initially identified in melanoma, in which it showed decreased expression^19^. *AIM2* consists of a HIN structural domain at the C-terminus and a PYD domain at the N-terminus and can identify double-strand DNA (dsDNA) of microbes or the host^20^. *AIM2* activates *CASP-1* through *ASC*-mediated junctional proteins to promote the maturation and release of IL-1β and IL-18 and to promote pyroptosis^21^. *AIM2* was originally regarded as a tumour suppressor because its inactivation or mutation was found in a variety of tumours, including endometrial, gastric, and colon cancers, but it was found to be overexpressed in oral, nasopharyngeal, and non-small-cell lung cancer^22^. Therefore, *AIM2* may play a unique role in different cancer types. Interestingly, in our study, *AIM2* seemed to be a cancer-promoting gene, as it was upregulated threefold in tumour tissues; however, it also contributed to prolonged patient survival because it was enriched in the low-risk group. Given the limited data from OC and the often conflicting results in different tumours, our results regarding *AIM2* provide some insights for further studies. Phospholipase C gamma 1 (*PLCG1*) is involved in the receptor tyrosine kinase (RTK)-mediated signal transduction pathway, thus affecting cell growth, differentiation, and apoptosis^23^. Recently, Kang et al.^24^ demonstrated that knockdown of *PLCG1* inhibited *GSDMD*-N-induced cell death and indicated that *PLCG1* could mediate the activity of *GSDMD* and pyroptosis. However, the relationships between *PLCG1*-mediated pyroptosis and tumour development remain largely unknown. We found that high *PLCG1* expression was connected with poor survival outcomes, which may be a result of its negative regulation of pyroptosis. *ELANE* is one of the major serine proteases secreted by neutrophils, and it activates proinflammatory cytokines such as *TNF-α*, *IL-1β*, and *IL-18*^25,26^, which are known to be pyroptosis promoters. Kambara et al.^27^ proved that *GSDMD* could be cleaved and activated by *ELANE* and induce neutrophils to undergo pyroptosis. The expression of *ELANE* was significantly higher while the neutrophil infiltration score was much lower in the high-risk group than in the low-risk group (in both the TCGA and GEO cohorts); these results may be because *ELANE* activates pyroptosis in neutrophils. *PJVK*, also known as *DFNB59*, is the only member of the gasdermin family that lacks the C-terminal domain, and it is not clear whether this protein can induce membrane perforation and execute pyroptosis^28^. As it is a member of the gasdermin family and has a complete N-terminal domain, we treated it as a “potential” pyroptosis-related gene. *PJVK* has been demonstrated to be associated with deafness, while its role in tumours has been little explored^29^. We found that *PJVK* was downregulated in tumour tissues, and its low expression predicted poor survival rates, indicating that it functioned as a tumour suppressor gene in this study. Further studies may focus on whether/how *PJVK* participates in pyroptosis and tumour suppression. *CASP3* exists as a non-activated zymogen in its normal state, but upon activation, it produces active executors that cleave structural and regulatory proteins in the nucleus and cytoplasm of cells, thereby regulating cell death, and it is recognized as a marker of apoptosis^30^. In 2017, Wang et al.^31^ discovered that *GSDME* was specifically cleaved by chemotherapeutic drug-activated *CASP3* to produce a membrane-penetrating *GSDME*-N fragment, which induced pyroptosis. *CASP3* was upregulated in patients with increased survival times in our analysis, and *CASP3* may be related to increased sensitivity to chemotherapeutic-drug induced pyroptosis. *CASP6* has been proven to modulate inflammasome activation (including activation of *NLRP3*, *ASC*, and *CASP1*) to promote GSDMD-induced pyroptosis^32^. In addition, *CASP6* also plays an important role in promoting apoptosis and necroptosis^33^, but the specific mechanisms by which it improves the survival rate of OC patients still need further exploration. *GSDMA* has a two-domain structure that can be self-inhibited, namely, the N-terminal structural domain can be inhibited by the C-terminal structural domain. The N-terminal domain can bind membrane lipids, phosphatidylinositol, and cardiolipin, forming pores in the cell membrane to trigger pyroptosis^8^. *GSDMA* acted as a cancer-promoting gene in our study due to its overexpression in OC tissues and its negative correlation with survival time. In summary, 5 genes (*CASP3*, *CASP6*, *AIM2*, *PLCG1*, and *ELANE*) in the prognostic model were proven to be pyroptosis promoters, and 2 genes (*PJVK* and *GSDMA*) were identified as possible pyroptosis executors. However, these promoters and executors were not all associated with better OC prognosis in our study. How these genes interact with each other during pyroptosis remains to be further investigated.

Until now, pyroptosis has not been fully studied, although certain similarities to apoptosis, as well as some crossovers in mechanisms, have been found. As tumours develop, multiple modes of cell death may coexist and interact with each other^34^. For example, 3 genes (*CASP3*, *CASP6*, and *PLCG1*) in our model are also known as key regulators in apoptotic pathways. Generally, apoptosis features an intact cell plasma membrane and no release of contents and does not directly cause inflammatory responses, while pyroptosis shows the opposite characteristics^35^. We analysed the DEGs between different risk groups and found that the DEGs were mainly involved in immune responses and inflammatory cell chemotaxis, indicating that dying cells induce intense inflammatory responses. Based on the results of our GO and KEGG analyses, it is reasonable to speculate that pyroptosis can regulate the composition of the tumour immune microenvironment.

The levels of key antitumour infiltrating immune cells were low, indicating an overall impairment of immune functions in the high-risk group in the TCGA cohort, and this conclusion was verified in the GEO cohort. Surprisingly, Treg cells were found in higher proportions in a low-risk group than in the high-risk group in our study, while they have been reported to suppress antitumour immunity and to be correlated with poor clinical outcomes in previous studies^36,37^. A possible reason for this discrepancy may be that Treg cells are essential in the tumour microenvironment to regulate the overactive inflammatory reactions caused by pyroptosis. In addition, in colon cancers, two main subtypes of Treg cells that have opposite roles in the regulation of the tumour microenvironment have been reported^38^; therefore, it is worth identifying the subtypes of Treg cells in OC. Except for the type II *IFN* response pathway, other immune pathways were poorly activated in the high-risk group in the two cohorts. Based on these findings, the poor survival outcome of high-risk OCs may be caused by decreased levels of antitumour immunity.

There is little current research on pyroptosis, especially on its mechanism in OC. Our study identified 2 genes in the gasdermin family that may be the executors of pyroptosis in OC and 5 genes that have the ability to regulate pyroptosis. We preliminarily studied the prognostic value of these pyroptosis-related genes and provided theoretical support for future research. However, due to a lack of data, we could not confirm whether these regulators (which have been reported in prior studies) also play corresponding roles in pyroptosis pathways in OC, and this question deserves further in-depth studies.

In summary, our study demonstrated that pyroptosis is closely connected to OC because most of the pyroptosis-related genes between normal and OC tissues were differently expressed. Moreover, the score generated from our risk signature based on 7 pyroptosis-related genes was an independent risk factor for predicting OS in both the TCGA and GEO cohorts. The DEGs between the low- and high-risk groups were associated with tumour immunity. Our study provides a novel gene signature for predicting the prognosis of OC patients and offers a significant basis for future studies of the relationships between pyroptosis-related genes and immunity in OC.

## Materials and methods

### Datasets

We obtained the RNA sequencing (RNA-seq) data of 379 OC patients and the corresponding clinical features from TCGA database on 30 November 2020 (https://portal.gdc.cancer.gov/repository). The RNA-seq data of 88 normal human ovarian samples were downloaded from the GTEx database (https://xenabrowser.net/datapages/). The RNA-seq data and clinical information of the external validation cohort were downloaded from the GEO database (https://www.ncbi.nlm.nih.gov/geo/, ID: GSE140082). The follow-up time of each participant in the GSE140082 cohort was up to 4 years, which was shorter than that in the TCGA cohort.

### Identification of differentially expressed pyroptosis-related genes

We extracted 33 pyroptosis-related genes from prior reviews^17–20^, and they are presented in Table S1. Due to the lack of normal ovarian tissue data in the TCGA cohort, we also considered GTEx data from 88 normal ovarian samples to identify the DEGs between normal and tumour tissues. The expression data in both datasets were normalized to fragment per kilobase million (FPKM) values before comparison. The “limma” package was used to identify DEGs with a *P* value <0.05. The DEGs are notated as follows: * if *P* < 0.05, ** if *P* < 0.01, and *** if *P* < 0.001. A PPI network for the DEGs was constructed with Search Tool for the Retrieval of Interacting Genes (STRING), version 11.0 (https://string-db.org/).

### Development and validation of the pyroptosis-related gene prognostic model

To assess the prognostic value of the pyroptosis-related genes, we further employed Cox regression analysis to evaluate the correlations between each gene and survival status in the TCGA cohort. To prevent omissions, we set 0.2 as the cut-off *P*-value, and 7 survival-related genes were identified for further analysis. The LASSO Cox regression model (R package “glmnet”) was then utilized to narrow down the candidate genes and to develop the prognostic model. Ultimately, the seven genes and their coefficients were retained, and the penalty parameter (*λ*) was decided by the minimum criteria. The risk score was calculated after centralization and standardization (applying the “scale” function in R) of the TCGA expression data, and the risk score formula was as follows: Risk Score= $\mathop {\sum}\nolimits_i^7 {Xi \times Yi}$ (*X*: coefficients, *Y*: gene expression level). The TCGA OC patients were divided into low- and high-risk subgroups according to the median risk score, and the OS time was compared between the two subgroups via Kaplan–Meier analysis. PCA based on the 7-gene signature was performed by the “prcomp” function in the “stats” R package. The “survival”, “survminer” and “timeROC” R packages were employed to perform a 3-year ROC curve analysis. For the validation studies, an OC cohort from the GEO database (GSE140082) was employed. The expression of each pyroptosis-related gene was also normalized by the “scale” function, and the risk score was then calculated by the same formula used for the TCGA cohort. By applying the median risk score from the TCGA cohort, the patients in the GSE140082 cohort were also divided into low- or high-risk subgroups, and these groups were then compared to validate the gene model.

### Independent prognostic analysis of the risk score

We extracted the clinical information (age and grade) of patients in the TCGA cohort and the age and International Federation of Gynaecology and Obstetrics (FIGO) stage data of patients in the GEO cohort. These variables were analysed in combination with the risk score in our regression model. Univariate and multivariable Cox regression models were employed for the analysis.

### Functional enrichment analysis of the DEGs between the low- and high-risk groups

OC patients in the TCGA cohort were stratified into two subgroups according to the median risk score. The DEGs between the low- and high-risk groups were filtered according to specific criteria (|log_2_FC| ≥ 1 and FDR < 0.05). Based on these DEGs, GO and KEGG analyses were performed by applying the “clusterProfiler” package. The “gsva” package was utilized to conduct the ssGSEA to calculate the scores of infiltrating immune cells and to evaluate the activity of immune-related pathways.

### Statistical analysis

**Fig. 8:**
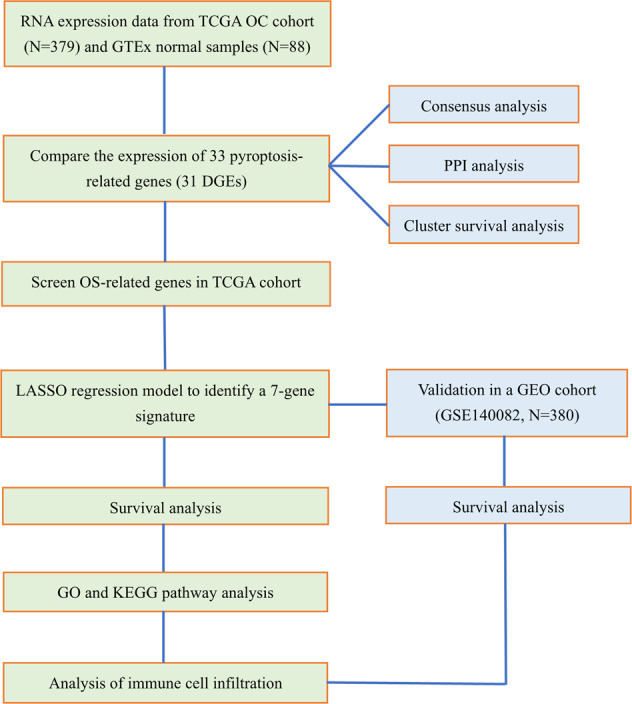
Workflow diagram. The specific workflow graph of data analysis.

Single-factor analysis of variance was applied to compare the gene expression levels between the normal ovarian and OC tissues, while the Pearson chi‐square test was used to compare the categorical variables. To compare the OS of patients between subgroups, we employed the Kaplan–Meier method with a two-sided log-rank test. To assess the independent prognostic value of the risk model, we used univariate and multivariate Cox regression models. When comparing the immune cell infiltration and immune pathway activation between the two groups, the Mann–Whitney test was used. All statistical analyses were accomplished with R software (v4.0.2). The overall flow diagram is shown in Fig. 8.

## Supplementary information

Table S1

Table S2

Table S3

## Data Availability

The data could be download at (https://portal.gdc.cancer.gov/, https://xenabrowser.net/, and https://www.ncbi.nlm.nih.gov/geo/; GSE140082) and the code used during the current study are available from the corresponding author on reasonable request.
